# Rapid Detection of Gut Microbial Metabolite Trimethylamine N-Oxide for Chronic Kidney Disease Prevention

**DOI:** 10.3390/bios11090339

**Published:** 2021-09-14

**Authors:** Yu-Chun Chang, Yi-Hsuan Chu, Chien-Cheng Wang, Chih-Hsuan Wang, You-Lin Tain, Hung-Wei Yang

**Affiliations:** 1 Institute of Medical Science and Technology, National Sun Yat-Sen University, Kaohsiung 804201, Taiwan; adamycchang@gmail.com; 2 Saint Dominic Catholic High School, Kaohsiung 802306, Taiwan; jolenechu@nsysumel.com (Y.-H.C.); pcjason11@gmail.com (C.-C.W.); 3 Department of Internal Medicine, Kaohsiung Armed Forces General Hospital, Kaohsiung 802301, Taiwan; wangchihhsuan@gmail.com; 4 Department of Pediatrics, College of Medicine, Kaohsiung Chang Gung Memorial Hospital, Chang Gung University, Kaohsiung 833401, Taiwan; 5 Institute for Translational Research in Biomedicine, College of Medicine, Kaohsiung Chang Gung Memorial Hospital, Chang Gung University, Kaohsiung 833401, Taiwan

**Keywords:** nanoenzyme, trimethylamine N-oxide (TMAO), gut microbiota, colorimetry, chronic kidney disease (CKD)

## Abstract

The gut microbiota plays a critical role in chronic kidney disease (CKD) and hypertension. Trimethylamine-N-oxide (TMAO) and trimethylamine (TMA) are gut microbiota-derived metabolites, and both are known uraemic toxins that are implicated in CKD, atherosclerosis, colorectal cancer and cardiovascular risk. Therefore, the detection and quantification of TMAO, which is a metabolite from gut microbes, are important for the diagnosis of diseases such as atherosclerosis, thrombosis and colorectal cancer. In this study, a new “colour-switch” method that is based on the combination of a plasma separation pad/absorption pad and polyallylamine hydrochloride-capped manganese dioxide (PAH@MnO_2_) nanozyme was developed for the direct quantitative detection of TMAO in whole blood without blood sample pretreatment. As a proof of concept, a limit of quantitation (LOQ) of less than 6.7 μM for TMAO was obtained with a wide linear quantification range from 15.6 to 500 μM through quantitative analysis, thereby suggesting potential clinical applications in blood TMAO monitoring for CKD patients.

## 1. Introduction

Chronic kidney disease (CKD) is a highly prevalent disease that affects nearly 10% of the world’s population [1]. There is increasing evidence that trimethylamine N-oxide (TMAO) is among the critical metabolites of gut bacteria, and it has been found to be associated with adverse cardiac events and CKD [2]. It is a key biomarker for a wide variety of human cardiac and kidney diseases [3] and was recently identified as a biomarker for colorectal cancer [4]. In people, TMAO is mostly eliminated from the body through urine and sweat [5]. Since the main clearance of TMAO from the body is through urine [6], a real clinical demand remains for new methods for rapidly detecting TMAO in serum or urine for the diagnosis and risk prediction of clinical diseases, such as CKD [7,8].

The commonly used methods for TMAO quantification are GC- and LC-MS/MS [9,10] and ^1^H NMR [11] techniques that generally require complicated and laborious procedures, long analysis times, specially trained personnel, and costly analytical instruments that are not widely available in clinical diagnostic laboratories. To address these issues, the development of a simple, inexpensive and rapid analytical method for TMAO detection is urgently needed. Wollenberger et al. reported an electrochemical biosensor for TMAO detection that uses TMAO reductase, namely, TorA, which specifically catalyses the reduction of TMAO to trimethylamine (TMA) [12], followed by the measurement of the resulting current using direct electrochemistry to quantify the concentration of TMAO [13]. However, its practical application has been limited due to the need to separate amine analytes and the lack of simplicity, efficiency, and portability of these reductions. Wang and coworkers explored a facile fluorescence assay for TMAO quantification on the basis of an indicator displacement assay (IDA) using water-soluble guanidinium-modified calix [5] arene (GC5A) as an artificial receptor [14]. However, its practical application has been limited because the indicator displacement may be affected by other substances in real urine samples.

Recently, researchers have increasingly paid attention to colourimetric array-based sensors in the biomedical biosensing field. Colourimetric sensors differ fundamentally from traditional electronic sensors in that the sensor signals depend not on physical sorption of analyte molecules (changes in the current) but instead on strong chemical interactions to change the colour (UV—vis absorption) [15]. Li and Suslick designed new nanostructured materials as colourimetric sensors for the specific detection of volatile amines [16]. However, the method requires an airtight container, the delivery of 13 inks on a polypropylene membrane, and the separation of other amine analytes, which limits its application for point-of-care (PoC) TMAO tests.

**Scheme 1:**
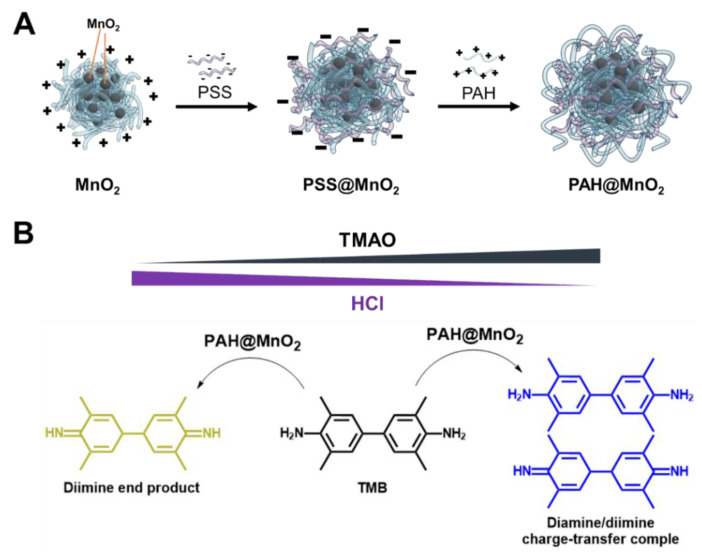
(**A**) An illustration of PAH@MnO_2_ synthesis; (**B**) the design and principle of colourimetric TMAO detection based on proton deposition of HCl by TMAO.

From the viewpoint of analytical science and with the above considerations, we demonstrated a new colourimetric assay for the rapid detection of TMAO with a simple procedure in which polyallylamine hydrochloride-capped manganese dioxide nanoparticles (PAH@MnO_2_ NPs) are used as reaction promoters and peroxidase mimics (Scheme 1A). We found that protons (from HCl) would be depleted during TMAO reduction, which would reduce the formation of diimine end products from TMB by PAH@MnO_2_ NPs to increase the absorbance intensity at 650 nm (A_650_) (Scheme 1B). The quantitative detection of TMAO was realised within the biological range without sample pretreatment, and excellent performance was demonstrated; thus, this assay has potential clinical applications in CKD PoC.

## 2. Experimental Section

### 2.1. Materials

All chemical reagents, which included trimethylamine-N-oxide (TMAO), polyallylamine hydrochloride (PAH, Mw 17,500 Da), polysodium 4-styrenesulfonate (PSS, Mw 70,000 Da), potassium permanganate, hydrogen chloride (HCl), sodium chloride (NaCl), 3,3′,5,5′-tetramethylbenzidine (TMB), and hydrogen peroxide (H_2_O_2_), were purchased from Sigma–Aldrich.

### 2.2. Preparation of PAH-Capped MnO_2_ NPs (PAH@MnO_2_ NPs)

PAH@MnO_2_ NPs were prepared by directly mixing aqueous solutions of KMnO_4_ and PAH. Briefly, 1 mL of PAH solution (1 mg/mL) that contained 0.1 M NaCl was mixed with 15 μL of KMnO_4_ solution (27.6 mg/mL), and the mixture was left for 20 min at room temperature until all permanganate was converted to MnO_2_. MnO_2_ formation was confirmed by observing with the naked eye a colour change to brown from purple. Subsequently, MnO_2_ NPs were added to PSS solution (1 mg/mL, 0.1 M NaCl) in equal volume and shaken for 10 min to form PSS-capped MnO_2_ NPs, which were centrifuged (12,000 rpm for 15 min) to remove the unreacted chemicals. Then, the precipitate was resuspended in PAH solution (1 mg/mL, 0.1 M NaCl) and shaken for 30 min. The mixture was washed three times with DI-H_2_O using centrifugation to obtain PAH@MnO_2_ NPs.

### 2.3. Stability Tests

HRP and PAH@MnO_2_ NPs were stored in a vacuum dry oven at 25 and 50 °C. After storage for various periods (1–60 days), the stored HRP and PAH@MnO_2_ NPs were used to catalyse H_2_O_2_/TMB and showed an absorption intensity of 450 nm (A_450nm_) after the addition of HCl. The activities of the stored HRP and PAH@MnO_2_ NPs were determined by comparing their A_450nm_ values with their initial A_450nm_ values at Day 0. All measurements were performed in triplicate for accurate calculations to develop a standard protocol.

### 2.4. pH Effect on the Peroxidation Activity

The peroxidation activities of HRP and PAH@MnO_2_ NPs towards H_2_O_2_/TMB at various pH values (pH 3 to pH 11) were analysed at 25 °C. The activities were determined by measuring A_450nm_ after the addition of HCl. All measurements were performed in triplicate for accurate calculations to develop a standard protocol.

### 2.5. Colourimetric Detection of TMAO

For the detection process, various concentrations of TMAO (100 μL), which ranged from 15.6 to 1000 μM, were mixed with 20 μL HCl and 20 μL PAH@MnO_2_ NPs for 3 min at room temperature. Subsequently, 20 μL of the above mixture was drawn out and mixed with 100 μL of TMB/H_2_O_2_ solution. Finally, the colour of the solution changed to light blue from yellow with increasing TMAO concentration, and the results were recorded by a SpectraMax M2 spectrometer to obtain A_650_. The A_650_ values were linearly proportional to the concentration of TMAO in a linear detection range. All experiments were conducted in sextuplicate for precise calculations to develop a standard protocol.

### 2.6. Preparation of an Artificial Urine Solution

An artificial urine solution was prepared according to previous reports [14,17], namely, by dissolving urea (9.1 g), sodium chloride (3.7 g), sodium phosphate (monobasic, 2.4 g), potassium chloride (2.2 g), creatinine (1.0 g) and BSA (25 mg) in 500 mL DI-H_2_O. The pH of the artificial urine solution was adjusted to pH 6.0 with HCl and stored at 4 °C. Subsequently, the artificial urine was spiked with various concentrations of TMAO (100, 250, and 500 μM) for recovery tests. The detection procedure was the same as in Section 2.5.

### 2.7. TMAO Rapid Detection in Plasma from CKD Rats

All animal studies were approved by the Institutional Animal Ethics Committee (IACUC) of Chang Gung Memorial Hospital (permit number 2020031602). For the samples, 16 female Sprague Dawley (SD) rats were purchased from BioLASCO Taiwan Co., Ltd. (Taipei, Taiwan).

At six weeks of age, rats of both sexes received a regular diet (Normal group, 8 rats) or a diet that was supplemented with 0.5% adenine (0.25 mg/kg/day) for three weeks (CKD group, 8 rats). The dose was selected based on our previous studies in rats [18]. At nine weeks of age, the rats were sacrificed to collect heparinised blood samples. The plasma TMAO concentration was determined by liquid chromatography-mass spectrometry (LC–MS) using previously described methods [19]. The TMAO concentration in the blood of each rat was also determined by the proposed colourimetric assay. First, a drop of blood was dropped on a plasma separation pad that covered an absorption pad. Then, the filtered solution (serum or plasma) was sucked into the absorbent pad and put into a well of a 96-well plate that contained 100 µL of H_2_O, 20 µL of HCl and 20 µL of PAH@MnO_2_ NPs at room temperature. Subsequently, 20 μL of the above mixture was drawn out and mixed with 100 μL of TMB/H_2_O_2_ solution. The obtained value of A_650_ was recorded to determine the TMAO concentration in the whole blood of normal/or CKD rats using the respective calibration curves for the colourimetric assay with the SpectraMax M2 spectrometer. Animal care and experiments were conducted according to established guidelines for the care and use of laboratory animals.

### 2.8. Statistics

The data are expressed as the mean ± SD on the basis of at least three independent experiments. Statistical analysis was performed using Student’s *t*-test. Differences were considered statistically significant if * *p* < 0.05.

## 3. Results and Discussion

### 3.1. Characterisation of the PAH@MnO_2_ NPs

**Figure 1:**
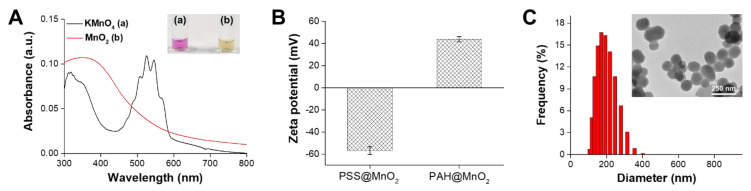
(**A**) UV–vis absorption spectra of KMnO_4_ and MnO_2_ in DI-H_2_O; the inset is a corresponding digital photo. (**B**) Zeta potential values of PSS@MnO_2_ and PAH@MnO_2_ (*n* = 3). (**C**) Dynamic light scattering (DLS) analysis results of PAH@MnO_2_ NPs; the inset shows the corresponding TEM.

The synthesis of PAH@MnO_2_ NPs was conducted according to a reported procedure with some modifications [20]. Typically, MnO_2_ NPs were prepared by reducing KMnO_4_ to MnO_2_ with cationic PAH. As shown in Figure 1A, the main peak of KMnO_4_ at 526/546 nm disappeared, but a new peak at 352 nm that corresponded to MnO_2_ NPs appeared after MnO_2_ formation, thereby demonstrating that KMnO_4_ was successfully reduced to form MnO_2_ with cationic PAH [21]. This result was also confirmed by a colour change (from purple to brown, Figure 1A inset). To stabilise MnO_2_, we deposited polyelectrolytes on the surface of MnO_2_. After deposition of PSS, the zeta potential of PSS@MnO_2_ was reduced to −56.8 ± 3.6 mV from 18.6 ± 4.6 mV because the attached PSS has a mass of carboxyl groups on the external surface. After self-assembly with PAH to form PAH@MnO_2_, the zeta potential further changed to 43.8 ± 2.3 mV, which enabled PAH@MnO_2_ to disperse in a water well (Figure 1B). The PAH@MnO_2_ NPs were stable and dispersed well in DI-H_2_O with an average diameter of 158.6 nm after PSS and PAH layer-by-layer assembly (Figure 1C). Transmission electron microscopy (TEM) images showed that the PAH@MnO_2_ NPs established prominent size uniformity and dispersibility in the form of a walnut-like shape (Figure 1C, inset).

### 3.2. Mechanism of TMAO Detection Using PAH@MnO_2_ NPs

**Figure 2:**
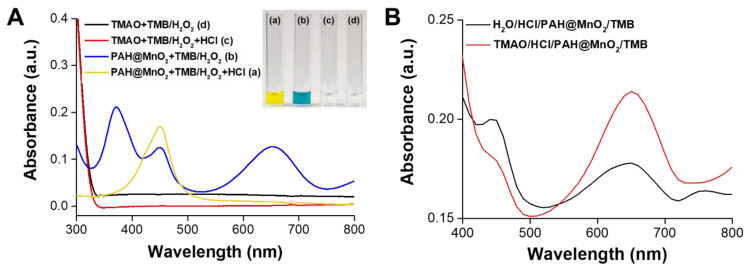
(**A**) UV-vis absorption spectra and visual colour changes of the catalysed oxidation of TMB in the absence or presence of PAH@MnO_2_; the inset is a corresponding digital photo; (**B**) UV-vis absorption spectra of the catalysed oxidation of TMB in the absence or presence of TMAO to prove the mechanism of proton depletion by TMAO.

In this study, we created a simple colourimetric assay for analysing TMAO by evaluating the oxidation level of TMB in the presence of H_2_O_2_ using PAH@MnO_2_ NPs as nanozymes. As shown in Figure 2A, neither TMAO nor HCl oxidised TMB in the presence of H_2_O_2_ to cause a colour change. In contrast, PAH@MnO_2_ NPs could oxidise TMB in the presence of H_2_O_2_ to form a diamine/diimine charge-transfer complex and induce a new peak at 650 nm (blue colour, curve (b). In the presence of HCl, the produced diamine/diimine charge-transfer complex further changed to a diimine end product. The main peak at 650 nm disappeared, but a new peak at 450 nm appeared (yellow colour, curve (a). The results demonstrated that the amount of HCl affected the ratio between the diamine/diimine charge-transfer complex and the diimine end product after TMB oxidation. To further prove that TMAO could deplete HCl, the change in A_650_ was investigated. As shown in Figure 2B, A_650_ increased and the absorbance intensity at 450 nm (A_450_) decreased in the presence of TMAO, thereby indicating that TMAO could deplete HCl to reduce the production of the diamine end product. Thus, the detection of TMAO is feasible using the developed colourimetric analysis.

### 3.3. Comparison of PAH@MnO_2_ and HRP in Terms of Peroxidation Activity and Stability

**Figure 3:**
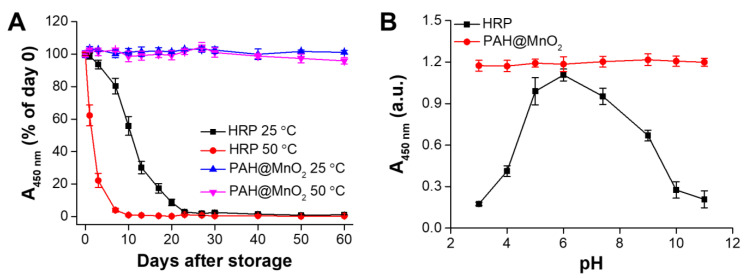
(**A**) The stabilities of HRP and PAH@MnO_2_ after storage at 25 and 50 °C for periods of 1–60 days. PAH@MnO_2_ showed no decrease in oxidisability towards H_2_O_2_/TMB after 60 days of storage at 50 °C. The values are means ± SD (*n* = 3); (**B**) the absorbances at 450 nm of HRP and PAH@MnO_2_ in the presence H_2_O_2_/TMB in PBS with various pH levels. The values are means ± SD (*n* = 3).

Most relevant studies in the literature have indicated the high peroxidation activity of an HRP weak acid environment towards TMB oxidation in the presence of H_2_O_2_; however, it may not be suitable for bioassays in any environment. Thus, a series of PBS solutions with pH values that varied from 3 to 11 were prepared to explore the effects of pH on HRP and PAH@MnO_2_. The results in Figure 3A indicate that the peroxidation activity of HRP is sensitive to pH, and the optimal result was obtained at a pH of 6; the activity significantly decreased when the pH was higher or lower than 6. In contrast, the peroxidation activity of PAH@MnO_2_ was not affected by the pH; the artificial enzyme PAH@MnO_2_ could effectively oxidise TMB in the presence of H_2_O_2_ to produce a consistent signal over a wide pH range from 3–11, thereby indicating that PAH@MnO_2_ is more suitable than HRP for the bioassay of clinical samples. The most common and severe problem that is encountered with enzyme-based biosensors is their lack of stability, which is due to the intrinsic nature of the enzyme. Thus, we evaluated the long-term stabilities of HRP and PAH@MnO_2_ after periods of storage of between 1 and 60 days at 25 and 50 °C; the responses were monitored by reacting TMB with HRP and PAH@MnO_2_ at each time point in the presence of H_2_O_2_. As shown in Figure 3B, HRP lost all its activity after 7 days of storage at 50 °C and did not maintain its activity beyond 23 days of storage at 25 °C. Impressively, PAH@MnO_2_ maintained its complete peroxidation activity without any decay, even when stored at 25 or 50 °C for 60 days. Based on these data, the peroxidase-mimicking PAH@MnO_2_ nanozyme that is described herein presents high oxidisation activity to TMB in the presence of H_2_O_2_, strong thermal stability, and low cost; hence, it is suitable for replacing HRP as the signal for colourimetric immunosensing.

### 3.4. Colourimetric Analysis of TMAO

**Figure 4:**
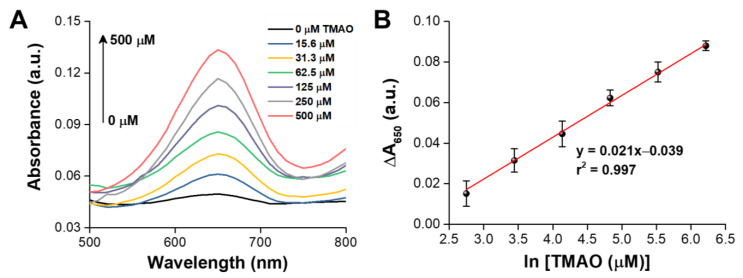
(**A**) Typical UV-vis absorption spectra of TMB for the detection of TMAO at various concentrations using the colour-switch method based on PAH@MnO_2_; (**B**) the linear calibration curve between A_650_ and the TMAO concentration. The values are means ± SD (*n* = 6).

To the best of our knowledge, LC–MS has been widely utilised to determine TMAO thus far with a wide linear detection range (10 μM~3000 μM) and a limit of quantitation (LOQ) of 5 μM; however, a stable isotopically labelled standard has been employed, which requires synthetic stable isotope markers, specially trained personnel, and specialised and costly analytical instruments, thereby limiting its clinical utilisation [22]. Consequently, semiquantifying TMAO in humour (especially in blood) has become an indispensable and urgent clinical task that facilitates the early diagnosis of CKD. In this study, we hypothesise that protons will be taken up during TMAO reduction to TMA in the presence of a catalyst that will reduce the formation of the diimine end product from TMB by PAH@MnO_2_ NPs to increase the value of A_650_. From the results in Figure 4A, A_650_ increased with increasing concentration of TMAO, and the standard curve was found to be linear in the range of 15.6 to 500 μM and a LOQ of 6.7 μM for TMAO when PAH@MnO_2_ NPs were used as the catalyst (r^2^ = 0.997; Figure 4B). Even though its linear detection range and LOQ are not superior to LC-MS, it has the advantage of simple, rapid and low-cost TMAO detection, which has potential clinical applications in CKD PoC.

### 3.5. Recovery of TMAO in Spiked Urine Samples

To prove the specificity and accuracy of our rapid detection of TMAO, we spiked various concentrations of TMAO into artificial urine, which ranged from 100 to 500 μM, and the A_650_ values were recorded by a spectrophotometer; the results are presented in Table 1. The recovery rates of TMAO were found to be acceptable and in the range of 97–102%, and the relative standard deviation was lower than 8%, thereby indicating that our TMAO rapid detection is accurate enough for TMAO detection without any interference.

### 3.6. TMAO Rapid Detection in Plasma from CKD Rats

**Figure 5:**
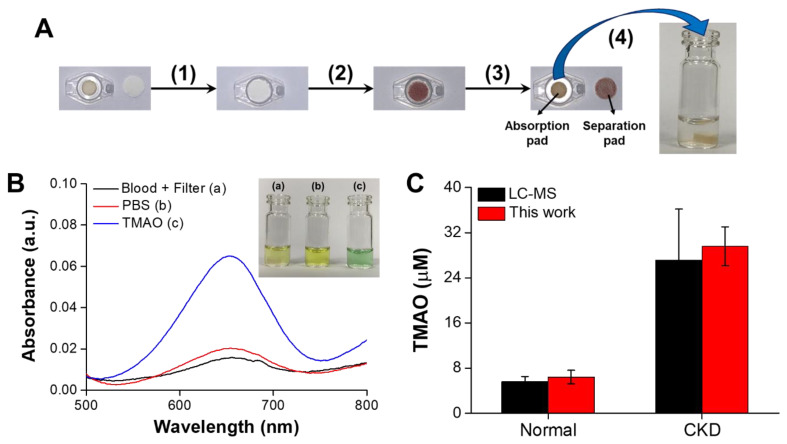
(**A**) The design and principle of colourimetric TMAO detection using whole blood based on the combination of a plasma separation pad as a erythrocyte trap and an absorption pad as serum/plasma trap to filter erythrocytes and haemachrome as follows: (1) the absorption pad is covered with the separation pad; (2) 50 μL of whole blood is dropped on the separation pad; (3) the serum/plasma is collected in the absorption pad and (4) the absorption pad with serum/plasma is placed into a glass vial for TMAO detection; (**B**) UV-vis absorption spectra and visual colour changes of the catalysed oxidation of TMB in two groups to confirm that the erythrocytes and haemachrome of whole blood could be filtered and that normal serum/plasma would not cause proton depletion, similar to PBS; the inset is a corresponding digital photo; (**C**) TMAO concentrations in 8 whole blood samples from normal rats and 8 whole blood samples from CKD that were measured using LC–MS and the proposed colourimetric assay for CKD monitoring. The values are means ± SD (*n* = 8).

Although it is easier to detect TMAO in a urine sample than in a blood sample, the concentration of TMAO in the urine of patients is easily affected by the amount of water they drink. However, if we were to directly measure the TMAO concentration in the blood using a colourimetric assay, the detection accuracy would be affected by the colour of the blood. In light of this, we designed a plasma separation pad that was coated with PEG8000 as an erythrocyte trap to retain and filter erythrocytes and haemachrome. Then, the filtered solution (possibly serum or plasma) was sucked into the absorbent pad for colourimetric assay of TMAO in fingertip blood (Figure 5A). Furthermore, we confirmed that the absorbent pad with serum/or plasma did not deplete the protons from HCl by observing a weak absorption peak at 650 nm, which was the same as for the PBS group. Conversely, a strong absorption peak at 650 nm was observed in the presence of 30 μM TMAO, thereby indicating that the protons from HCl were indeed depleted by TMAO to reduce the formation of the diimine end product from TMB by PAH@MnO_2_ NPs (Figure 5B). The results also demonstrated that the proposed colourimetric assay can be used for simple and rapid TMAO detection in fingertip blood. Finally, the feasibility and performance of the proposed colourimetric TMAO assay in CKD rats without sample pretreatment were studied. First, a drop of whole blood (~20 µL) from a CKD rat was dropped onto a plasma separation pad that covered an absorption pad to prevent colour interruption in the proposed colourimetric assay. Then, the absorption pad was put into a well of a 96-well plate that contained 100 µL of H_2_O, 20 µL of HCl and 20 µL of PAH@MnO_2_ NPs at room temperature. Subsequently, 20 μL of the above mixture was drawn out and mixed with 100 μL of TMB/H_2_O_2_ solution. A total of 16 whole blood samples (8 from normal rats and 8 from CKD rats) were used to assess the feasibility of our PAH@MnO_2_ NP-based colourimetric TMAO assay. Furthermore, the results were compared with those that were obtained using LC–MS, and no significant difference was found between them (Figure 5C). The results indicated that the accuracy of the proposed colourimetric assay for TMAO detection is comparable to that of LC–MS, but it is simpler and faster for daily CKD monitoring.

To understand whether the metabolites in the blood after eating or taking medication will affect the accuracy of TMAO detection using the proposed colourimetric assay, we detected the TMAO concentrations in blood from the rats that had been given 0.1 mL PBS (three rats), ascorbic acid (200 mg/mL, three rats), glucose (200 mg/mL, three rats), and sodium thiosulfate (200 mg/mL, three rats) for 24 h. The results demonstrated no significant difference between four groups, indicating that the metabolites in blood after diet or medication would not affect the accuracy of the proposed colourimetric assay for TMAO detection (Figure S1).

## 4. Conclusions

We have established a proton depletion method for the “colour-switch” sensing and quantitative assay of TMAO in whole blood via the PAH@MnO_2_ NPs nanozyme. To accurately determine concentrations of TMAO in the low µM range for practical diagnostic purposes, a wide linear calibration range of TMAO was successfully established. The feasibility was confirmed by detecting TMAO in whole blood samples from normal rats and CKD rats. In comparison to the previous methods (LC–MS), this proposed colourimetric assay provides a low-cost, easy-to-operate, label-free, rapid and sensitive method for TMAO determination, which may offer an alternative for TMAO detection in clinical studies. This method shows promise for application in tracking TMAO in blood and studying CKD progression in humans in the future.

## Figures and Tables

**Table 1:** Determination of spiked TMAO in artificial urine samples using the proposed colourimetric assay, which is based on the PAH@MnO_2_ nanozyme.

Sample	Spiked Concentration (μM)	Detected (μM)	Recovery (%)
Artificial urine	100	101.7 ± 7.2	101.7 ± 7.2
	300	303.4 ± 8.9	101.1 ± 3.0
	500	487.4 ± 8.7	97.5 ± 1.7

## Data Availability

Data is contained within the article.

## Supplementary Materials

The following are available online at https://www.mdpi.com/article/10.3390/bios11090339/s1, Scheme S1: (**A**) An illustration of PAH@MnO_2_ synthesis; (**B**) the design and principle of colourimetric TMAO detection based on proton deposition of HCl by TMAO, Figure S1: TMAO concentrations in whole blood from the rats after diet or medication for 24 h using the proposed colourimetric assay.
