# Supplementary material for: Rapid Detection of Gut Microbial Metabolite Trimethylamine N-Oxide for Chronic Kidney Disease Prevention
Source: Biosensors (Basel). 2021 Sep 14;11(9):339. doi: 10.3390/bios11090339 (PMC8469701; doi:10.3390/bios11090339)
Supplement: Supplementary file 1 [file biosensors-11-00339-s001.zip › biosensors-1369055-sup.pdf]

biosensors

MDPI

Article

Rapid Detection of Gut Microbial Metabolite Trimethylamine N-oxide for Chronic Kidney Disease Prevention

Yu-Chun Chang [1], Yi-Hsuan Chu [2], Chien-Cheng Wang [2], Chih-Hsuan Wang [3], You-Lin Tain [4,5,*], Hung-Wei Yang [1,*]

1 Institute of Medical Science and Technology, National Sun Yat-Sen University, Kaohsiung 804201, Taiwan; adamycchang@gmail.com
2 Saint Dominic Catholic High School, Kaohsiung 802306, Taiwan; jolenechu@nsysumel.com (Y.-H.C.); pcjason11@gmail.com (C.-C.W.)
3 Department of Internal Medicine, Kaohsiung Armed Forces General Hospital, Kaohsiung 802301, Taiwan; wangchihhsuan@gmail.com
4 Department of Pediatrics, College of Medicine, Kaohsiung Chang Gung Memorial Hospital, Chang Gung University, Kaohsiung 833401, Taiwan
5 Institute for Translational Research in Biomedicine, College of Medicine, Kaohsiung Chang Gung Memorial Hospital, Chang Gung University, Kaohsiung 833401, Taiwan
* Correspondence: tainyl@cgmh.org.tw (Y.-L.T.); howardyang@mail.nsysu.edu.tw (H.-W.Y.)

Additional Figure.

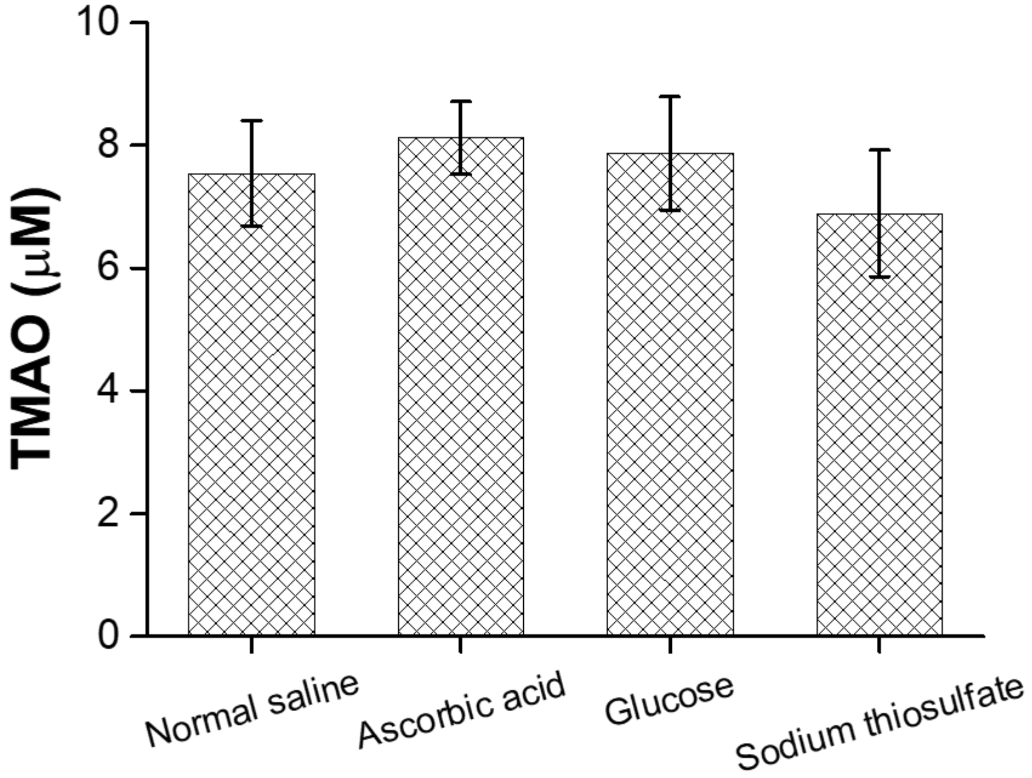

Figure S1. TMAO concentrations in 3 whole blood samples from normal rats injected with normal saline, 3 whole blood samples from normal rats injected with ascorbic acid (200 mg/mL), 3 whole blood samples from normal rats injected with glucose (200 mg/mL), and 3 whole blood samples from normal rats injected with sodium thiosulfate (200 mg/dL) that were measured using the proposed colorimetric assay. The values are means ± SD (n = 3).
